# Supplementary material for: Biosynthesizing structurally diverse diols via a general route combining oxidative and reductive formations of OH-groups
Source: Nat Commun. 2022 Mar 24;13:1595. doi: 10.1038/s41467-022-29216-5 (PMC8948231; doi:10.1038/s41467-022-29216-5)
Supplement: Supplementary file 1 — Supplementary Information [file 41467_2022_29216_MOESM1_ESM.pdf]

Biosynthesizing structurally diverse diols *via* a general route combining oxidative and reductive formations of OH-groups

Liu *et al.*

1.MPO

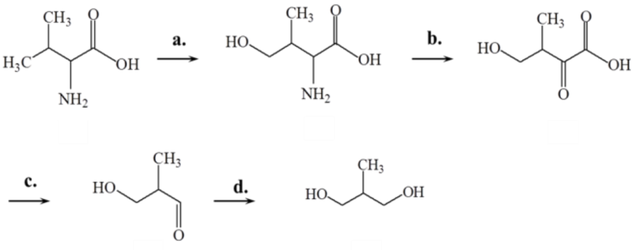

2.IPDO

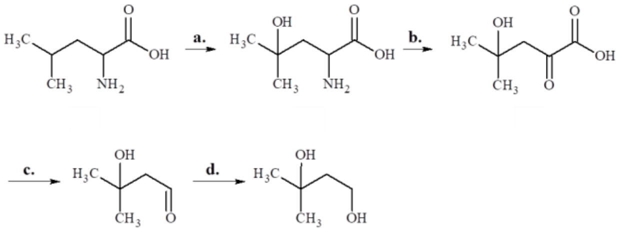

3.1,3-BDO

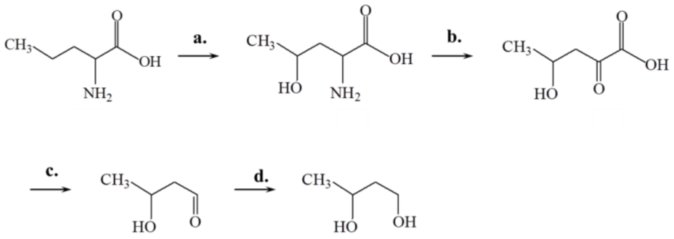

4.1,3-PTD

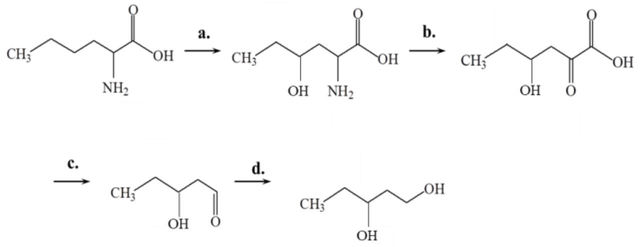

5.2E-1,4-PDO

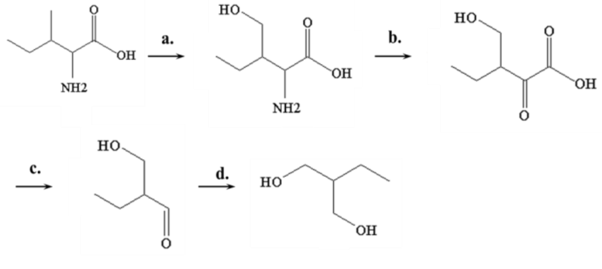

Supplementary Figure 1. Reaction schemes of the formation of MPO, IPDO, 1,3-BDO, 1,3-PTD and 2E-1,4-PDO produced through the proposed general pathway. “a”: hydroxylation reaction; “b”: deamination reaction; “c”: decarboxylation reaction; “d”: reduction reaction.

6.2M-1,3-BDO

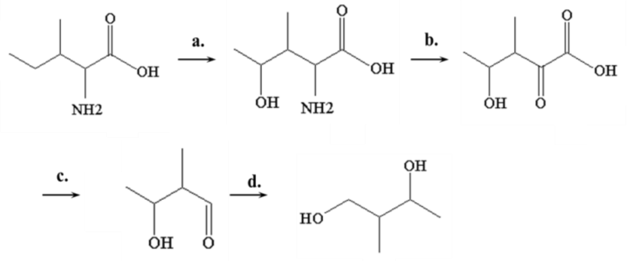

7.2M-1,4-BDO

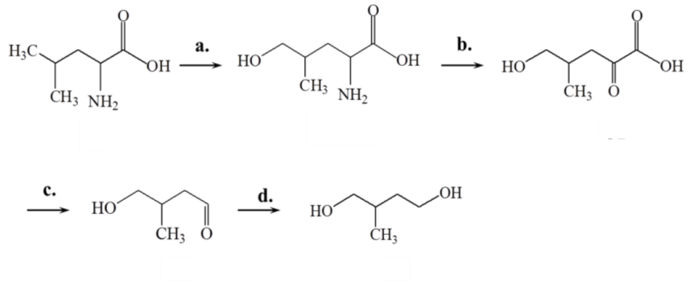

8.1,4-BDO

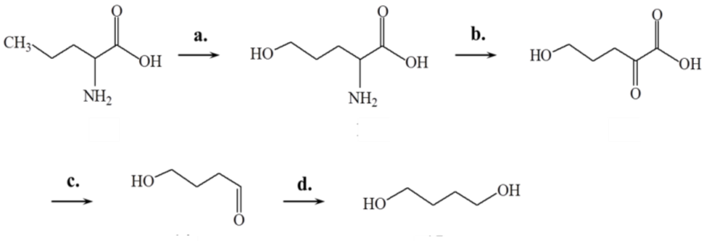

9.1,4-PTD

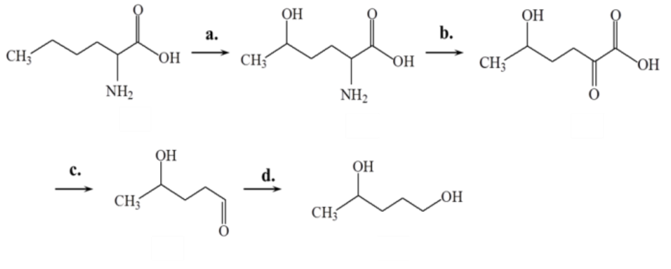

10.1,3-PDO

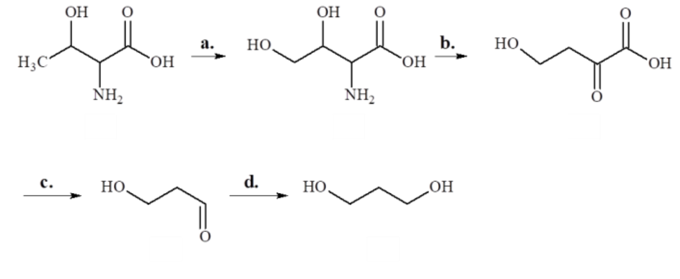

Supplementary Figure 2. Reaction schemes of the formation of 2M-1,3-BDO, 2M-1,4-BDO, 1,4-BDO, 1,4-PTD and 1,3-PDO produced through the proposed general pathway. “a”: hydroxylation reaction; “b”: deamination reaction; “c”: decarboxylation reaction; “d”: reduction reaction.

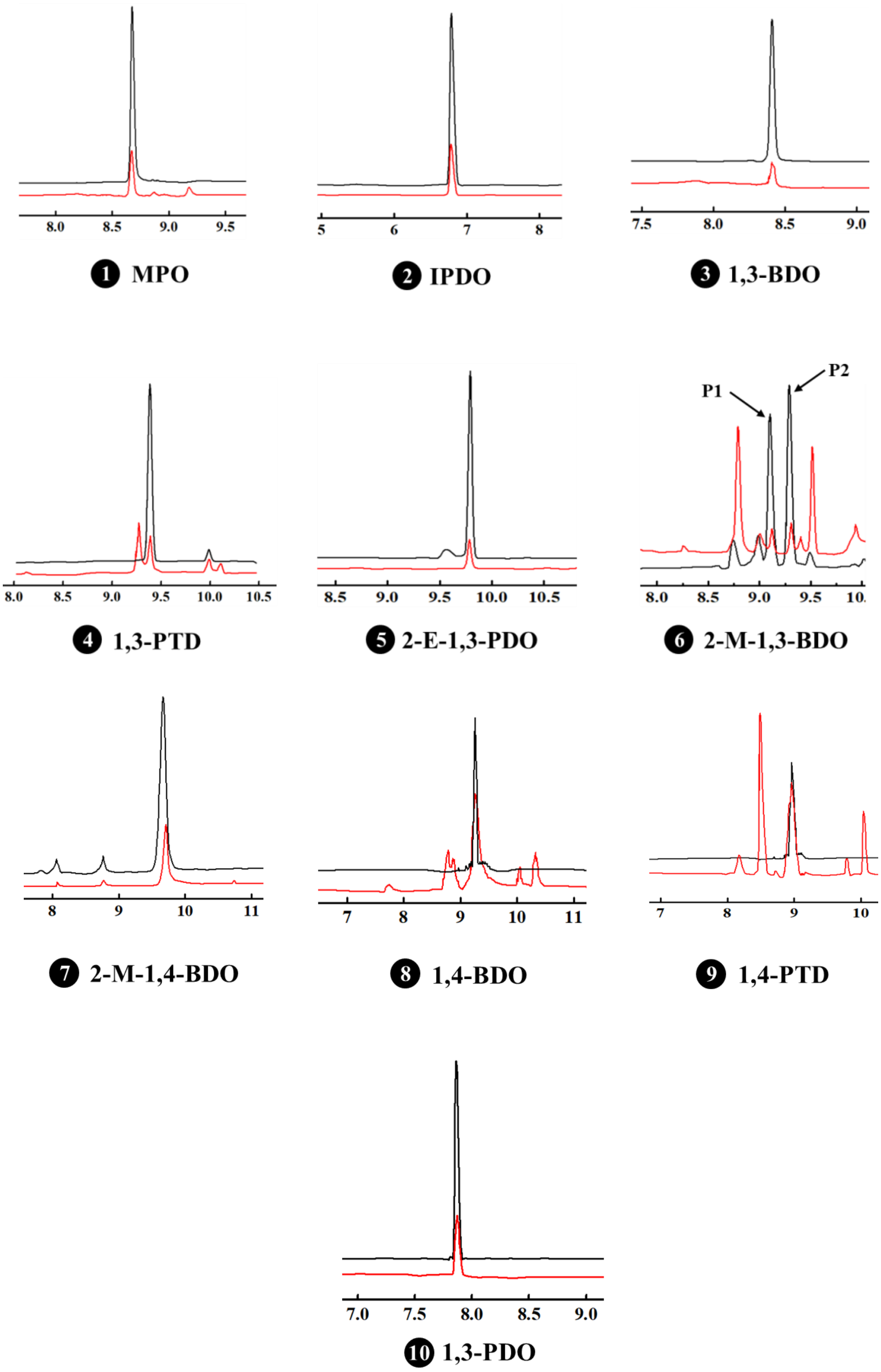

Supplementary Figure 3. Identification of the product diols by GC-MS analysis. Black lines represent total ion chromatographs (TIC) of purchased diol standards (100 mg/L) and red lines represent TICs of real samples. Two peaks (P1 and P2) were found on the TIC of both the standard and the real sample of 2M-1,3-BDO whose mass spectra are almost identical. The samples of IPDO, 2-E-1,3-PDO and 2-M-1,4-BDO were diluted by 10-fold before GC analysis. The samples of 1,4-BDO and 1,4-PTD were concentrated by 10-fold before GC analysis. The numbers of the diols are in line with those in Figure 2a.

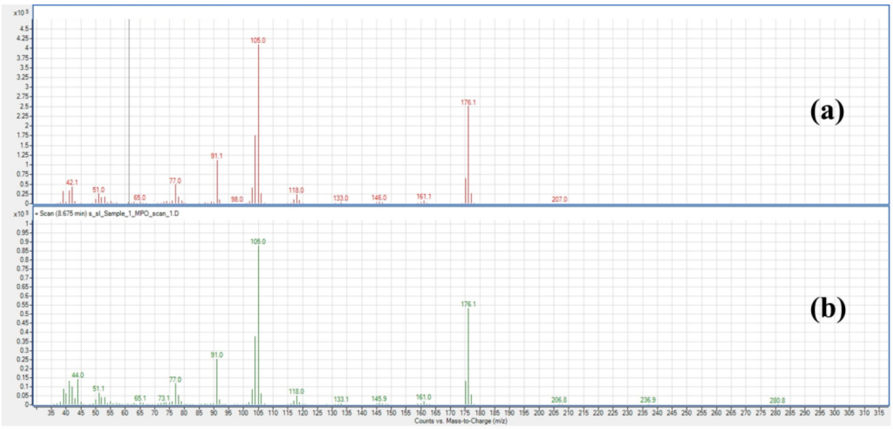

❶ **MPO**

Supplementary Figure 4. Mass spectrums of MPO. (a) MPO standard. (b) MPO in samples.

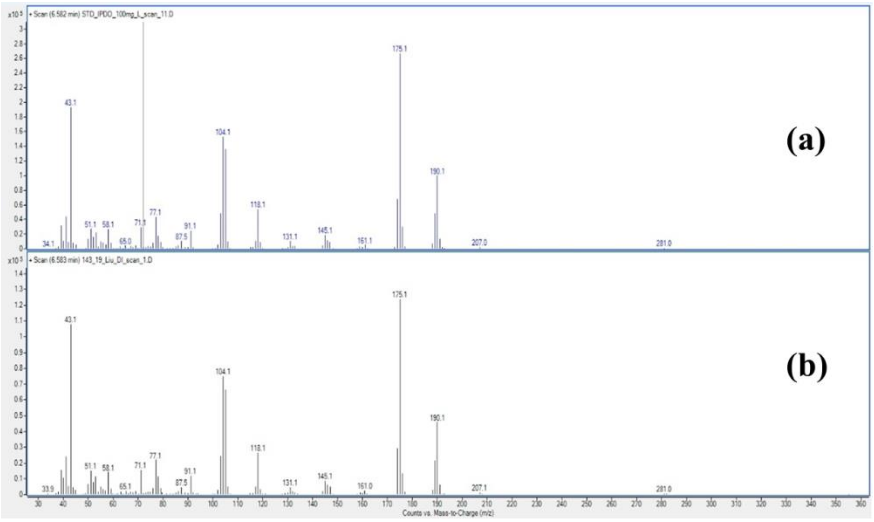

❷ IPDO

Supplementary Figure 5. Mass spectrums of IPDO. (a) IPDO standard. (b) IPDO in samples.

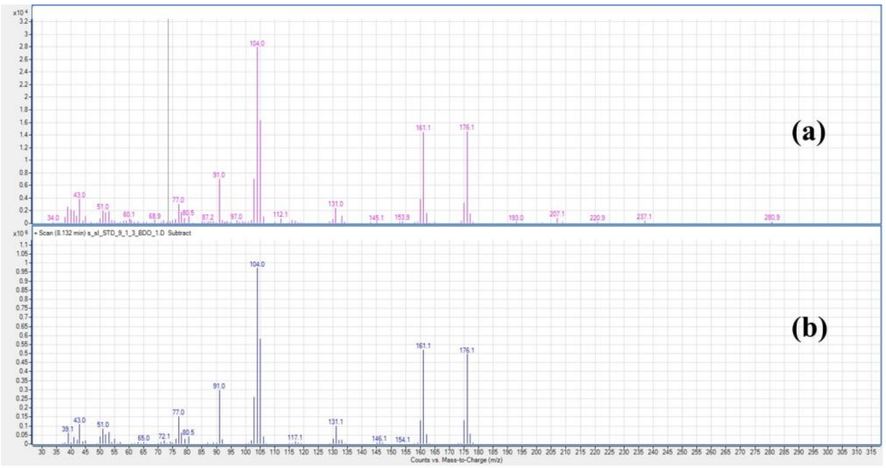

❸ **1,3-BDO**

Supplementary Figure 6. Mass spectrums of 1,3-BDO. (a) 1,3-BDO standard. (b) 1,3-BDO in samples.

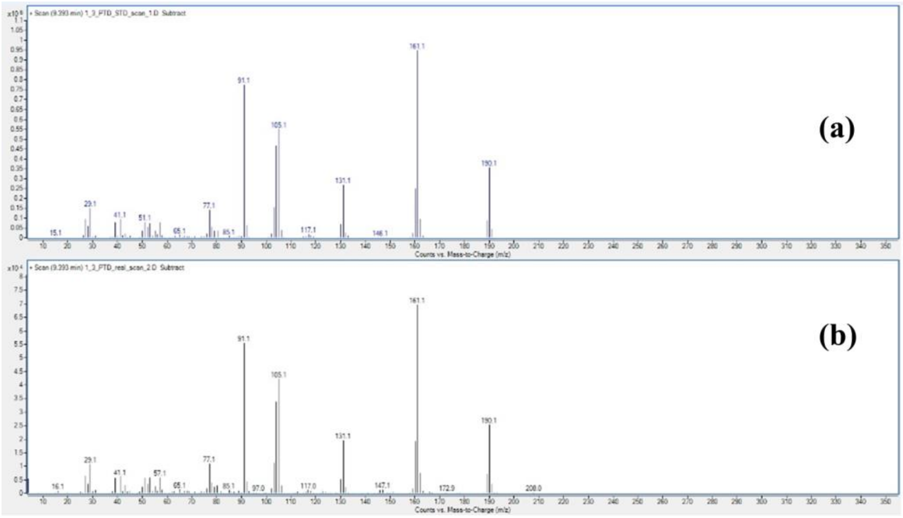

❹ **1,3-PTD**

Supplementary Figure 7. Mass spectrums of 1,3-PTD. (a) 1,3-PTD standard. (b) 1,3-PTD in samples.

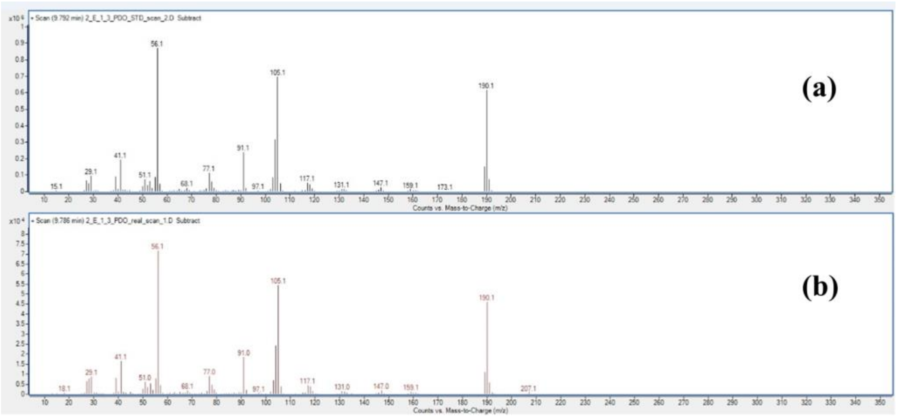

❺ **2-E-1,3-PDO**

Supplementary Figure 8. Mass spectrums of 2-E-1,3-PDO. (a) 2-E-1,3-PDO standard. (b) 2-E-1,3-PDO in samples.

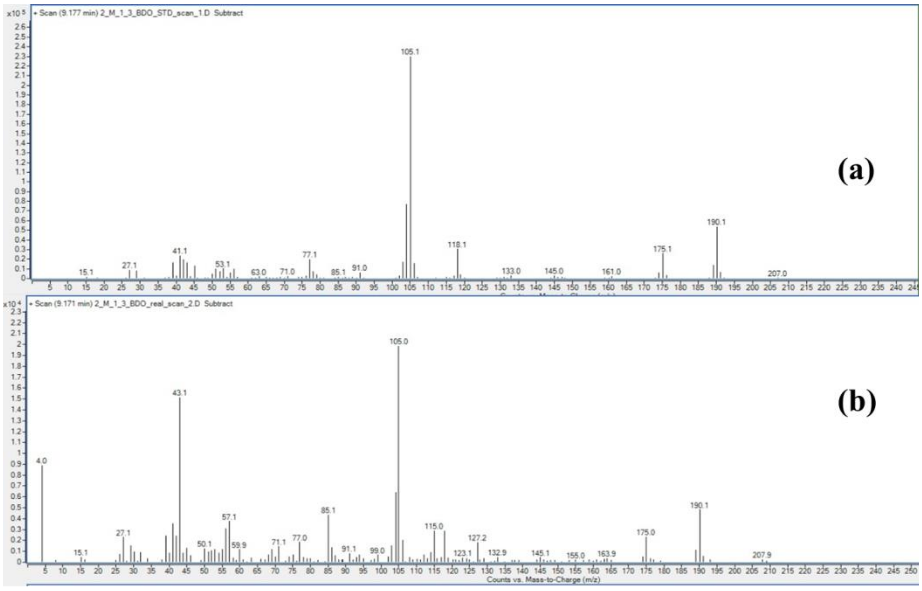

❻ **2-M-1,3-BDO-P1**

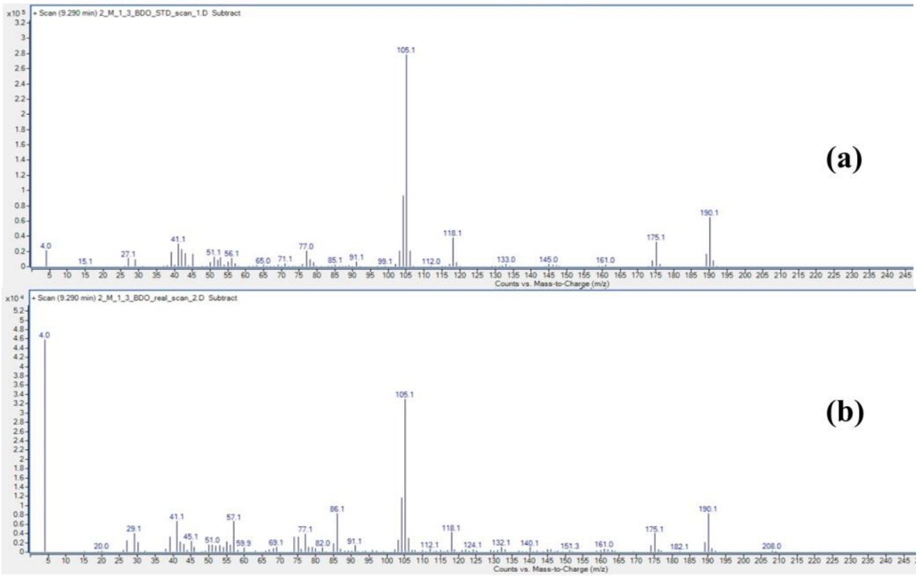

❻ **2-M-1,3-BDO-P2**

Supplementary Figure 9. Mass spectrums of 2-M-1,3-BDO. (a) 2-M-1,3-BDO standard. (b) 2-M-1,3-BDO in samples. Two peaks (P1 and P2) were found on the TIC of both the standard and the real sample of 2-M-1,3-BDO.

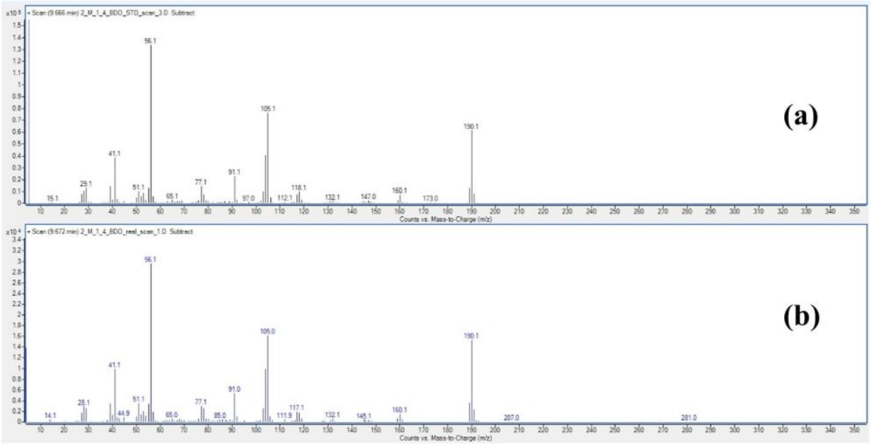

❼ **2-M-1,4-BDO**

Supplementary Figure 10. Mass spectrums of 2-M-1,4-BDO. (a) 2-M-1,4-BDO standard. (b) 2-M-1,4-BDO in samples.

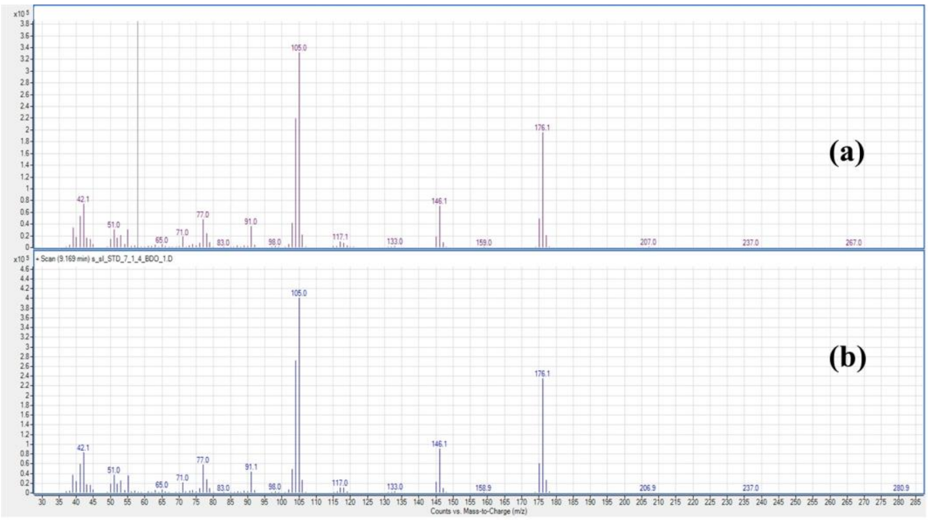

❽ **1,4-BDO**

Supplementary Figure 11. Mass spectrums of 1,4-BDO. (a) 1,4-BDO standard. (b)1,4-BDO in samples.

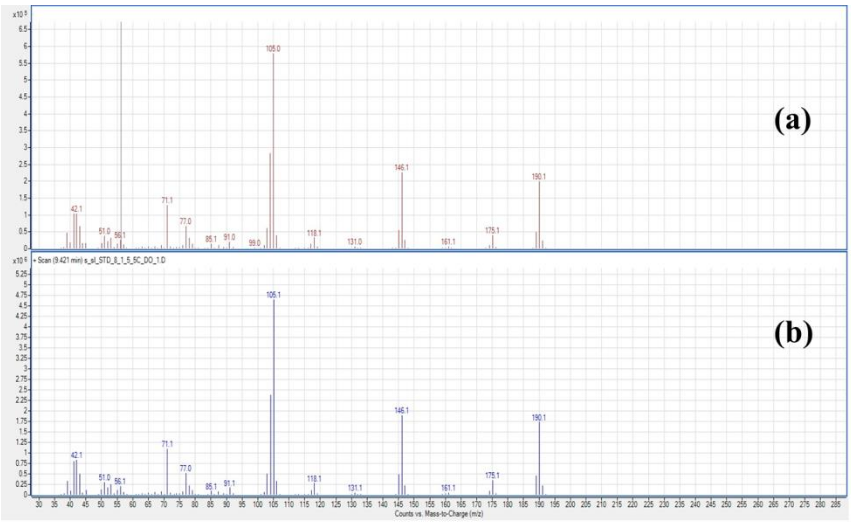

9 1,4-PTD

Supplementary Figure 12. Mass spectrums of 1,4-PTD. (a) 1,4-PTD standard. (b) 1,4-PTD in samples.

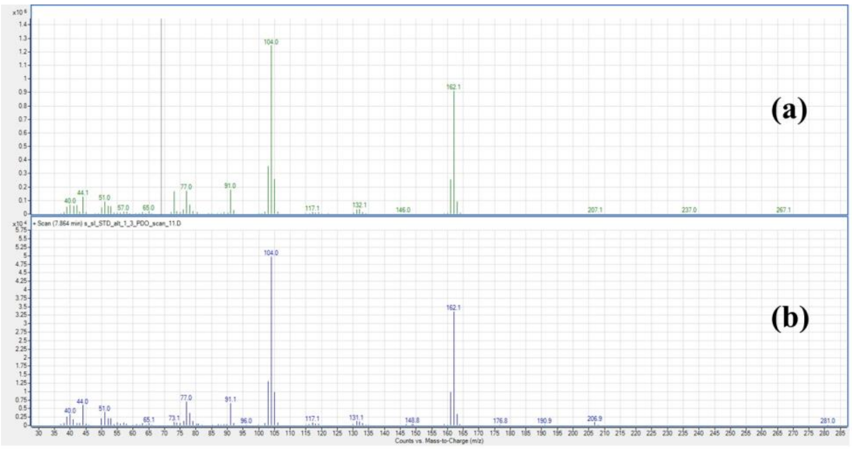

❿ **1,3-PDO**

Supplementary Figure 13. Mass spectrums of 1,3-PDO. (a) 1,3-PDO standard. (b) 1,3-PDO in samples.

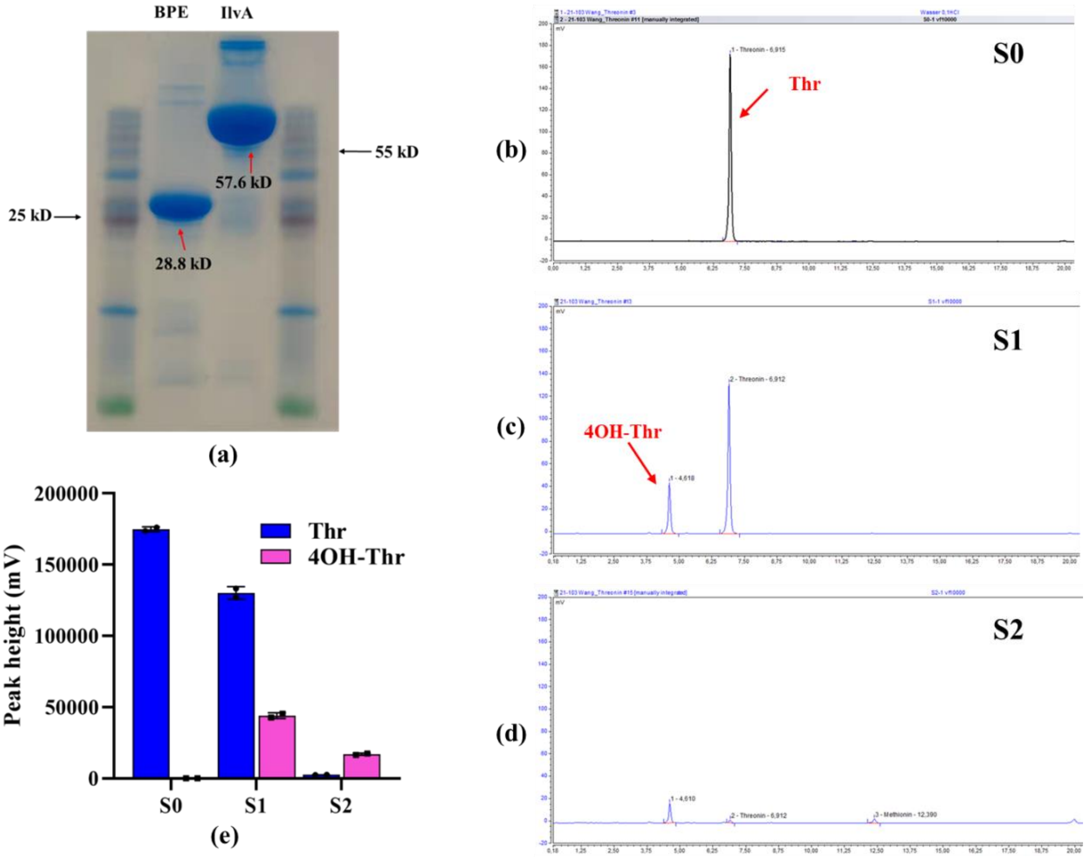

Supplementary Figure 14. *In vitro* verification of the specific activity of threonine deaminase IlvA on 4-hydroxyl-threonine. We carried out an in vitro enzymatic assay to verify the specific activity of IlvA on 4-hydroxy-threonine. Since 4-hydroxy-threonine is not commercially available, threonine hydroxylase BPE is used to synthesize 4-hydroxy-threonine from threonine. Both threonine hydroxylase BPE and threonine deaminase IlvA were separately ligated into pET-28a vector and transformed into *E. coli* BL21(DE3) strain. The two strains were induced by IPTG and cultivated at 30 ^{o}C overnight to overexpress BPE and IlvA, followed by purification by affinity chromatography on a His SpinTrap column (Supplementary Figure 3a.). To verify the activity of IlvA on 4-hydroxy-threonine, we set one control group S0 and two experimental groups S1 and S2. The components of the three groups are as follow: S0: 20 mM threonine + reaction buffer; S1: Adding 10 μM BEP in S0, reaction for 16h; S2: Adding 10 μM IlvA in S1, reaction for 8h. As can be seen from the results of HPLC analysis, when compared with the chromatograph of S0 (Supplementary Figure 3b), a new peak appears in the chromatograph of S1 (red arrow in Supplementary Figure 3c, retention time: 4.618 min), accompanied with the decrease of threonine, which can only be 4-hydroxy-threonine, the product of threonine hydroxylation. After the addition of threonine deaminase IlvA, the area of this peak in S2 drops to about 38% (Supplementary Figure 3c-e), clearly indicating that IlvA has activity on 4-hydroxy-threonine. It is noteworthy that the amount of threonine in S2 drops to less than 2% (Supplementary Figure 3d-e) which is mainly due to the fact that threonine as the natural substrate of IlvA is deaminated as well. For e, the average and s.d. of two biologically independent experiments are shown. Source data are provided as a Source Data file.

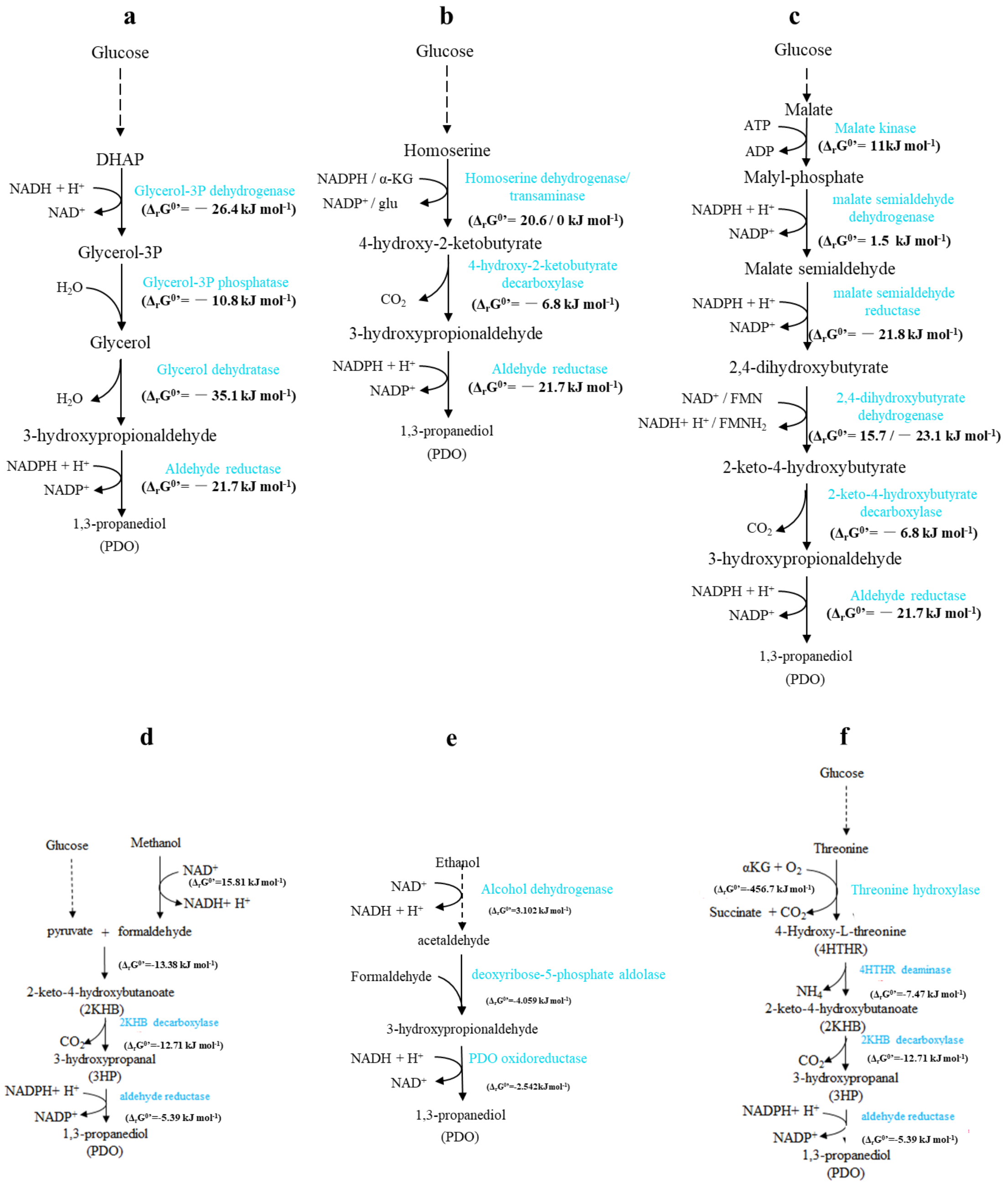

Supplementary Figure 15. Thermodynamic profiles of six artificial pathways for 1,3-PDO production. (a) 1,3-PDO is produced by integrating two naturally occurring pathways that convert glucose to glycerol and subsequently to 1,3-PDO, respectively[1]. (b) 1,3-PDO is synthesized via extending the homoserine catabolism[2]. (c) 1,3-PDO is formed via extending the malate catabolism[3]. (d) 1,3-PDO is produced by incorporating methanol into the formation of the appealing intermediate HOBA and finally 1,3-PDO[4]. (e) 1,3-PDO is produced by using ethanol as the substrate[5]. (f) The 1,3-PDO synthetic route proposed in this study. The thermodynamic profiles of the heterologous reaction steps involved in these five pathways and the novel 1,3-PDO biosynthetic pathway proposed in this study (f) were analyzed and compared.

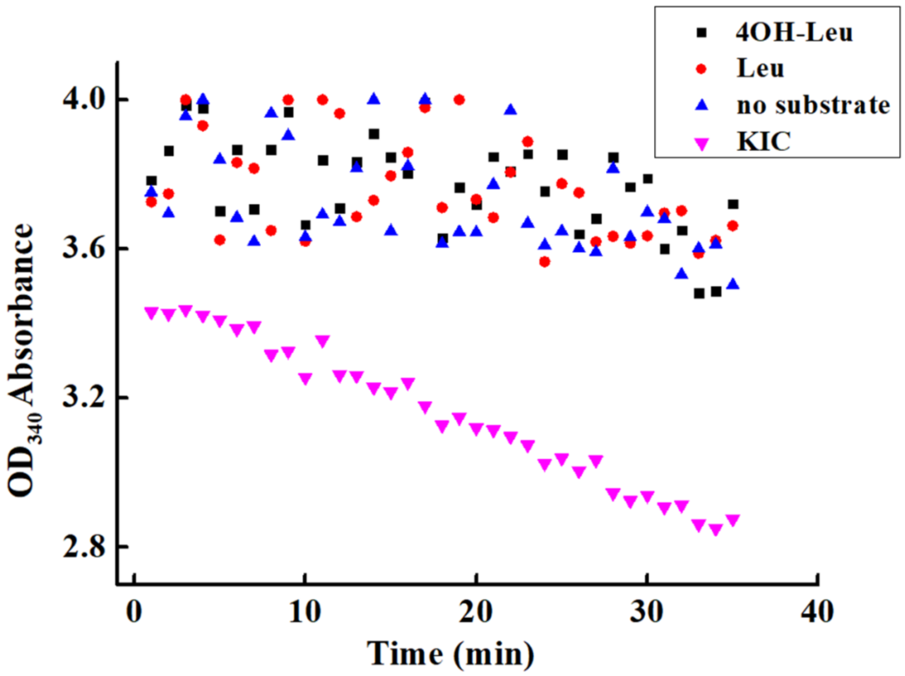

(a)

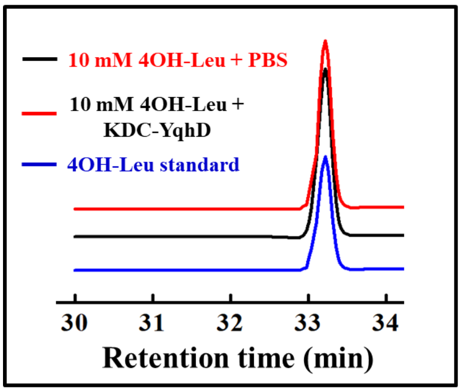

(b)

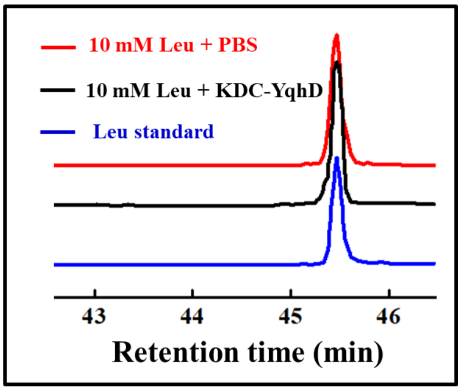

(c)

Supplementary Figure 16. Determination of KDC and YqhD activities towards 4OH-Leu and leucine. (a) Enzymatic activity results of KDC and YqhD on 4OH-Leu and leucine. A reaction system using KIC as precursor was used as the positive control, while a reaction system without the addition of precursor was the negative control. Results indicated that KDC and YqhD showed excellent activity on KIC but no activity on leucine and 4OH-Leu. (b, c) HPLC quantification of 4OH-Leu (b) and leucine (c) also confirmed that there were negligible changes in the concentrations of 4OH-Leu and leucine when comparing the reaction systems with (black) or without (red) KDC and YqhD. Source data are provided as a Source Data file.

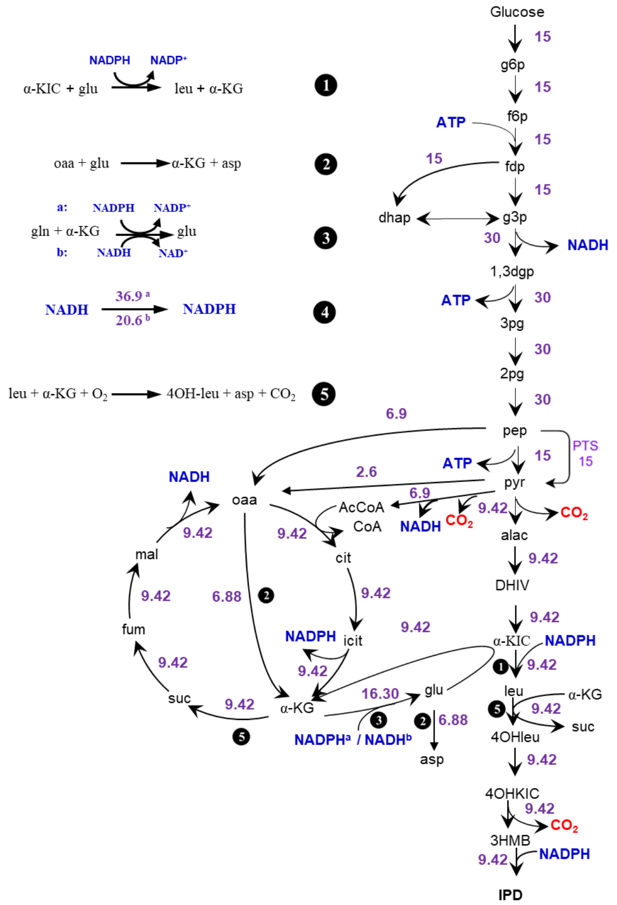

Supplementary Figure 17. Optimal metabolic flux distribution for IPDO production in *E. coli* iY75_1357 model. According to our flux balance analysis (FBA) result, glutamate maintains a balance by two degradation reactions (reaction 1 and 2) and one synthesis reaction (reaction 3). For reaction 1, the transamination reaction catalyzed by branched-chain-amino-acid aminotransferase converts α-ketoisocaproate to leucine. This reaction requires glutamate as the amino donor and costs one NADPH. In another degradation reaction (reaction 2), aspartate aminotransferase catalyzes the synthesis of aspartate and α-KG from glutamate and oxaloacetate. The synthesized α-KG, in turn, participates in the supply of glutamate through the catalysis of glutamate synthase (reaction 3), which utilizes either NADPH or NADH as reducing power. Besides, the balance of the two reducing powers is maintained by transhydrogenases (reaction 4). The metabolic flux of this reaction is dependent on whether glutamate dehydrogenase is NADPH (a) or NADH-dependent (b). Abbreviations: Glu, glucose; G-6P, Glucose-6-phosphate; F-6P, Fructose-6-phosphate; F-1,6P, Fructose-1,6-bisphosphate; G3P, Glyceraldehyde-3-phosphate; 3PG, Glycerate-3-phosphate; 2PG, Glycerate-2-phosphate; PEP, Phosphoenolpyruvate; PYR, Pyruvate; Acetyl-CoA, Acetyl Coenzyme A; OAA, Oxalosuccinate; Cit, Citric acid; Cis-aco, Cis-aconitase; ICit, Isocitrate; Oxa, Oxalosuccinate; AKG, α-ketoglutarate; SucCoA, Succinyl-CoA; Suc, Succinate; Fum, Fumarate; Mal, Malate; PGluc, 6-phosphate-glucose lactone; Ru5P, Ribulose-5-phosphate; Xu5P, Xylulose-5-phosphate; R5P, Ribose-5-phosphate; S7P, Sedoheptulose-7-phosphate; E4P, Erythrose-4-phosphate; DHIV, α, β-Dihydroxyisovalerate; KIC, α-Ketoisocaproate; 3HMB, 3-hydroxy, 3-methyl,butyraldehyde; 4OHleu, 4-hydroxyleucine; 4OHKIC, 4-hydroxy, 4-methyl pentanoic acid. IPD, IPDO.

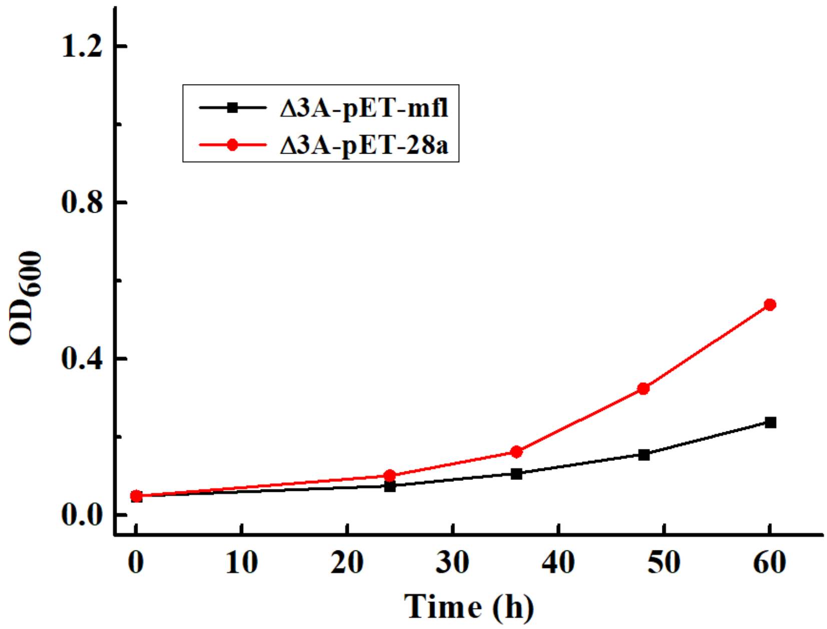

Supplementary Figure 18. Growth curves of Δ3A strain (*E. coli* BL21 Δ*sucA*Δ*aceA*Δ*putA* (DE3)(pLysS)) carrying pET28a (Δ3A-pET-28a) or pET-mfl (Δ3A-pET-mfl) in M9 medium. The initial OD was 0.05. Source data are provided as a Source Data file.

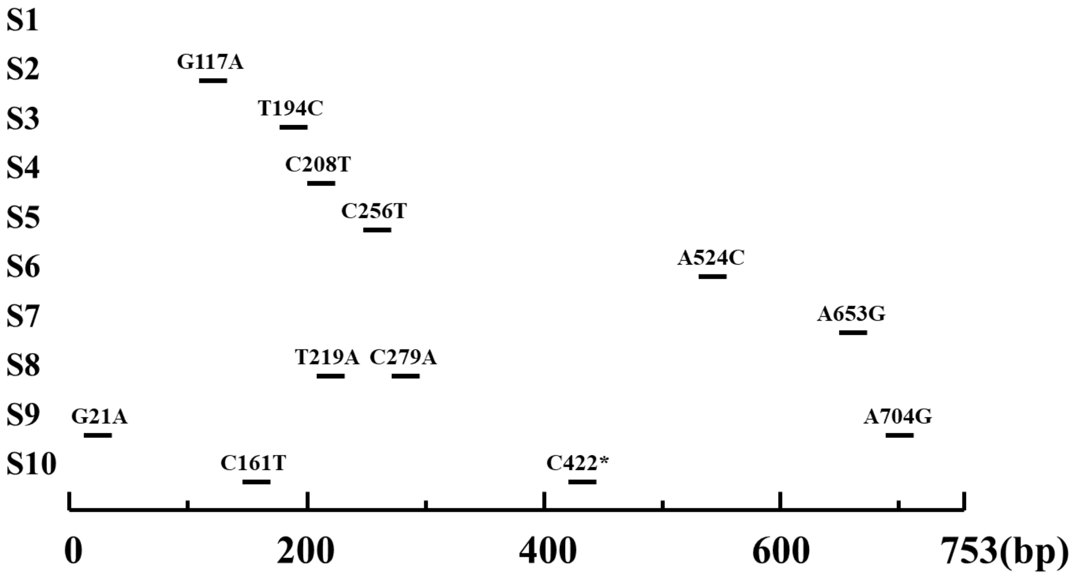

Supplementary Figure 19. Distribution of mutation sites in ten MFL mutants picked up from the library. The quality of the MFL mutant library was assessed before screening for mutants with higher activities using the strain Δ3A. Sequencing results of 10 colonies randomly picked from the library showed that nine in the ten mutants contained at least one site mutation. Among them, six contain one mutation site and three contain two mutation sites. The site mutations are distributed randomly throughout the MFL sequence, representing no bias toward any specific sequence space. This indicates that the quality of the mutant library was satisfactory and can be used for the subsequent screening of MFL mutants. Source data are provided as a Source Data file.

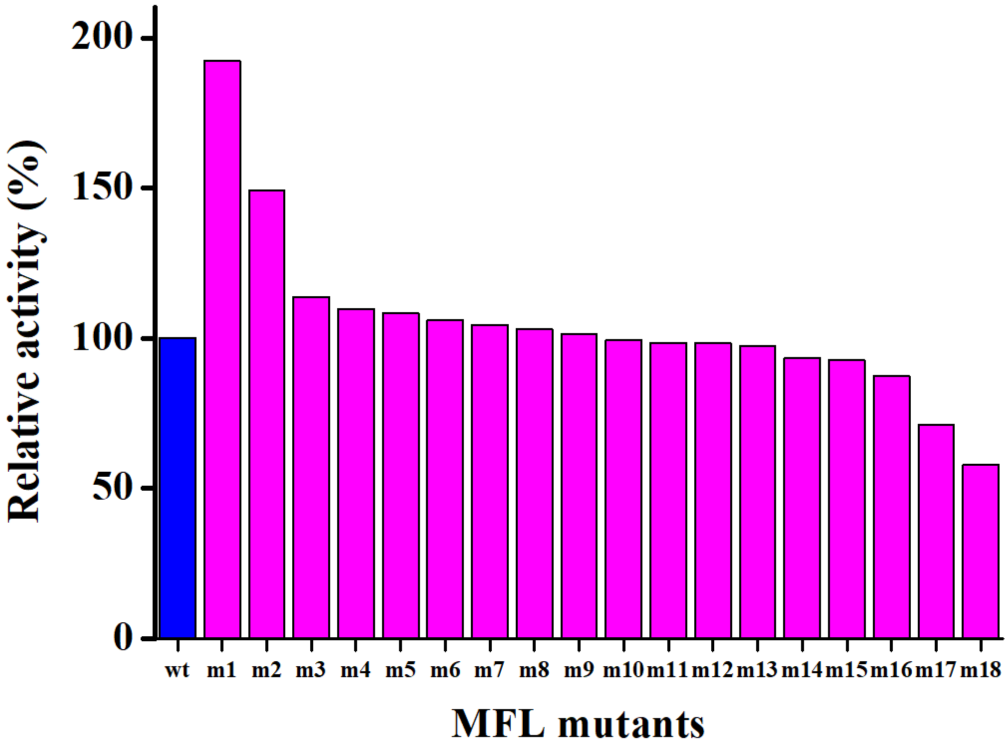

Supplementary Figure 20. Enzymatic activity of wild type hydroxylase MFL (MFL_{wt}) and its random mutants selected from M9 agar plates supplemented with 4 g/L yeast, 0.2 g/L leucine, 0.2 g/L α-KG and 0.1 mM IPTG. Source data are provided as a Source Data file.

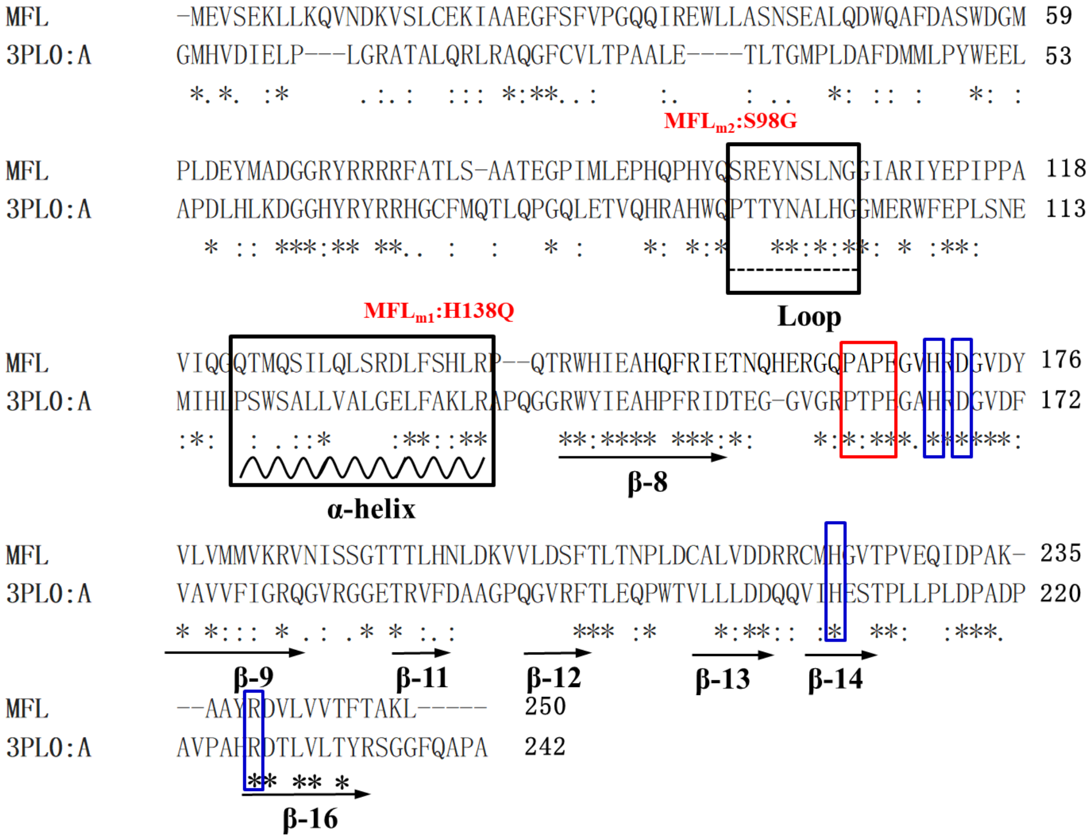

Supplementary Figure 21. Sequence alignment of MFL and MpDO (PDB code 3PL0:A). Site mutations (red underlined) in MFL_{m1} and MFL_{m2} are highlighted in red. Conserved residues involving in binding Fe^{2+} and α-KG (red frame) and a ^{159}PxPE^{162} motif (blue frame) are marked. The β-sheets that constitute the cup-shaped β-sandwich are marked with black arrows.

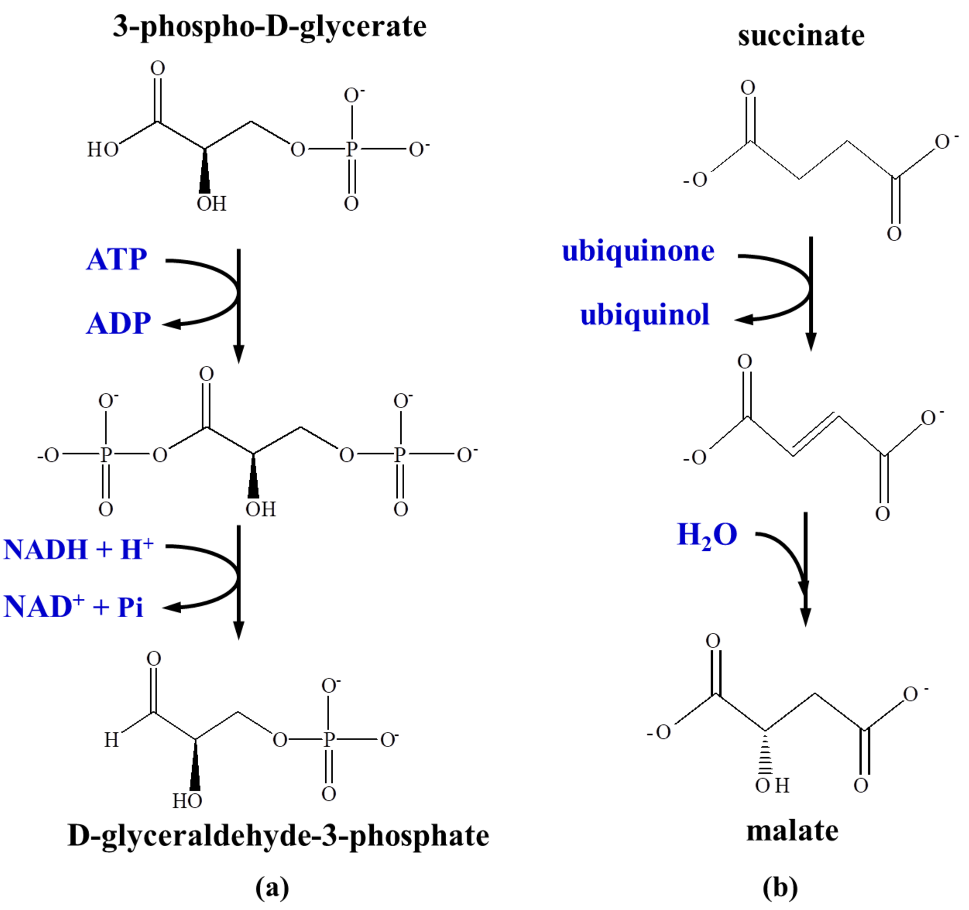

Supplementary Figure 22. Two strategies that organism employ to overcome the thermodynamic barriers in redox reactions. (a) Reduction of 3-phospho-D-glycerate to D-glyceraldehyde 3-phosphate in gluconeogenesis. (b) Reduction of succinate to malate in the TCA cycle.

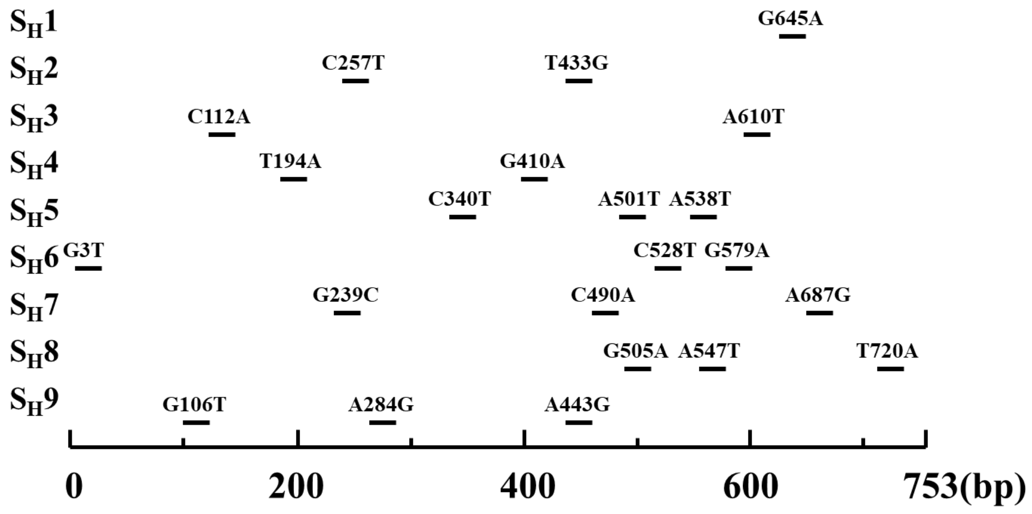

Supplementary Figure 23. Distribution of mutation sites in nine MFL mutants picked up from the library with higher mutation frequency. "H" means mutant library with higher mutation frequency. Source data are provided as a Source Data file.

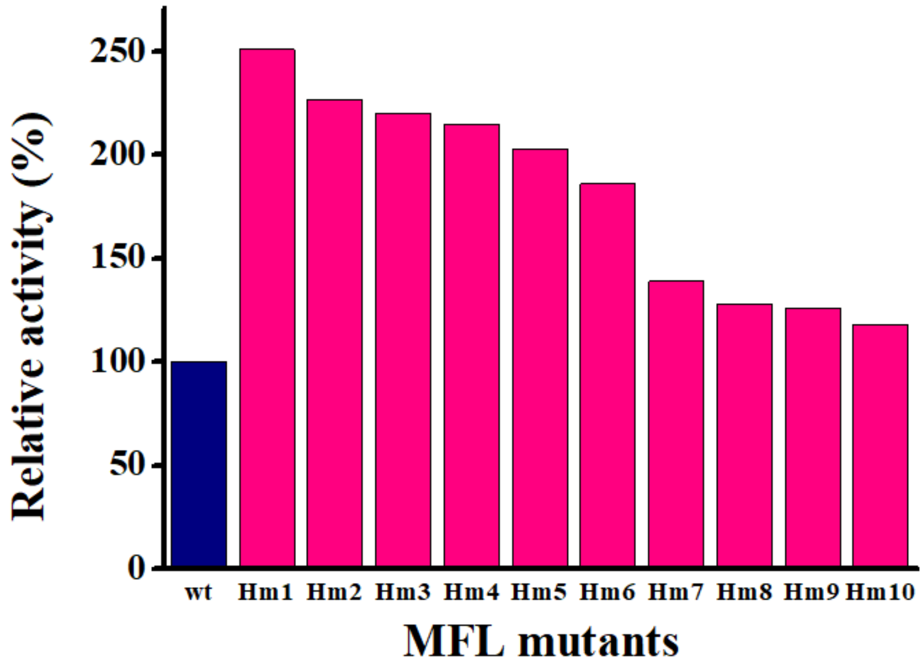

Supplementary Figure 24. Enzymatic activity of the wild type hydroxylase MFL (MFL_{wt}) and its random mutants picked up from the newly constructed library with higher mutation frequency. "H" means mutant library with higher mutation frequency. Source data are provided as a Source Data file.

Supplementary references

1. Hartlep, M. *et al*. Study of two-stage processes for the microbial production of 1,3-propanediol from glucose. *Applied Microbiology and Biotechnology* **60**, 60-66 (2002).
2. Chen, Z. *et al*. Protein design and engineering of a *de novo* pathway for microbial production of 1,3-propanediol from glucose. *Biotechnology Journal* **10**, 284-289 (2015).
3. Frazao, C. J. R. *et al*. Construction of a synthetic pathway for the production of 1,3-propanediol from glucose. *Scientific Reports* **9**, 11576 (2019).
4. Wang, C. *et al*. An aldolase-catalyzed new metabolic pathway for the assimilation of formaldehyde and methanol to synthesize 2-keto-4-hydroxybutyrate and 1,3-propanediol in *Escherichia coli*. *ACS Synthetic Biology* **8**, 2483-2493 (2019).
5. Meng, H. *et al*. An aldolase-based new pathway for bioconversion of formaldehyde and ethanol into 1,3-propanediol in *Escherichia coli*. *ACS Synthetic Biology* **10**, 799-809 (2021).
