# Supplementary material for: Biosynthesizing structurally diverse diols via a general route combining oxidative and reductive formations of OH-groups
Source: Nat Commun. 2022 Mar 24;13:1595. doi: 10.1038/s41467-022-29216-5 (PMC8948231; doi:10.1038/s41467-022-29216-5)
Supplement: Supplementary file 3 — Description of Additional Supplementary Files [file 41467_2022_29216_MOESM3_ESM.pdf]

File name: Supplementary Data 1.

Description: Strains and plasmids used in this study.

File name: Supplementary Data 2.

Description: Primers used in this study.
